# Time-Restricted Eating Improves Glycemic Control in Patients with Type 2 Diabetes: A Meta-Analysis and Systematic Review

**DOI:** 10.3390/ijms26157310

**Published:** 2025-07-29

**Authors:** Taegwang Nam, Hyeongbin Oh, Anna Kim, Yongtaek Oh

**Affiliations:** 1 College of Korean Medicine, Woosuk University, Jeonju 54986, Republic of Korea; skaxorhkd12@naver.com (T.N.); 0224ohb@naver.com (H.O.); 2 KM Data Division, Korea Institute of Oriental Medicine, Daejeon 34054, Republic of Korea

**Keywords:** time-restricted eating, diabetes mellitus, glycemic control, HbA1c, fasting glucose, time in range, meta-analysis

## Abstract

Time-restricted eating (TRE), a dietary strategy that aligns food intake with circadian rhythms, has emerged as a promising non-pharmacological approach for improving glycemic control in patients with type 2 diabetes. This systematic review and meta-analysis evaluated the effects of TRE on glycemic outcomes by analyzing eight randomized controlled trials involving 312 participants with type 2 diabetes or impaired fasting glucose. Meta-analyses of six eligible studies demonstrated that TRE significantly reduced fasting glucose (mean difference [MD]: −0.74 mmol/L; 95% CI: −1.13 to −0.36) and glycated hemoglobin (ΔHbA1c) (MD: −0.11%; 95% CI: −0.15 to −0.07) and increased time in range (TIR) for blood glucose (MD: +10.51%; 95% CI: 6.81 to 14.21). Improvements in fasting glucose and HbA1c were modest but consistent, while the increase in TIR showed no between-study heterogeneity, suggesting a robust and reproducible benefit of TRE on glycemic stability. These findings support the clinical feasibility and effectiveness of TRE as a dietary intervention in diabetes management. However, further high-quality trials with standardized protocols and longer follow-up are needed to confirm long-term efficacy and inform guidelines.

## 1. Introduction

Diabetes mellitus is a chronic metabolic disorder characterized by persistent hyperglycemia resulting from defects in insulin secretion, insulin action, or both. Based on its pathophysiology, diabetes is classified into type 1 diabetes (T1D) and type 2 diabetes (T2D) [1,2,3]. T1D primarily involves autoimmune destruction of pancreatic β-cells, whereas T2D is mainly driven by insulin resistance and a relative deficiency in insulin secretion. Despite these differences, both forms share key metabolic abnormalities, including impaired postprandial glucose regulation and increased hepatic glucose production [1,2,3].

The global prevalence of diabetes continues to rise. As of 2022, more than 11% of South Korean men were diagnosed with diabetes, and approximately one-third of men and women over the age of 70 were affected [4]. Globally, 1 in 11 people live with diabetes, and its prevalence has steadily increased over the past 30 years, ranking it as the ninth leading cause of death worldwide [1]. Diabetes is also a major contributor to serious complications such as cardiovascular disease, stroke, dementia, and peripheral neuropathies, highlighting the need for sustained and effective disease management [5,6,7,8,9].

Dietary intervention is a cornerstone of diabetes management, significantly influencing weight control, glycemic regulation, insulin sensitivity, and overall metabolic balance [10]. Recently, beyond conventional dietary strategies, time-restricted eating (TRE)—an approach that limits food intake to a specific time window each day—has emerged as a promising non-pharmacological intervention. TRE is designed to align food consumption with circadian rhythms, thereby improving metabolic homeostasis, promoting fat oxidation during fasting periods, and enhancing insulin responsiveness [11,12,13,14,15,16,17,18].

Several randomized controlled trials (RCTs) and case reports involving individuals with metabolic syndrome or diabetes have demonstrated potential benefits of TRE, including reductions in fasting glucose, improvements in insulin sensitivity, and weight loss [16,19,20,21,22,23,24,25]. However, high-level evidence from systematic reviews and meta-analyses is still lacking. Existing studies show inconsistency in results, involve small sample sizes, and vary in intervention protocols, making it difficult to draw firm clinical conclusions regarding the efficacy of TRE.

Therefore, this study aims to systematically review and conduct a meta-analysis of RCTs evaluating the effects of TRE on glycemic control in patients with type 1 and type 2 diabetes. Specifically, we quantitatively analyzed the effects of TRE on fasting glucose, glycated hemoglobin (HbA1c), and time in range (TIR). Other outcomes such as insulin resistance indices and body weight were reviewed narratively. By synthesizing the current evidence, this study seeks to support dietary strategy development in diabetes care and provide foundational data for future clinical research.

## 2. Materials and Methods

### 2.1. Protocol and Registration

The protocol for this systematic review was registered in PROSPERO (CRD420251050341) and it is available at https://www.crd.york.ac.uk/PROSPERO/view/CRD420251050341 (accessed on 27 May 2025).

### 2.2. Data Sources and Search Strategy

A comprehensive literature search was conducted across six English and Korean databases: the Cochrane Central Register of Controlled Trials (via Cochrane Library), MEDLINE (via PubMed), EMBASE (via Elsevier, Amsterdam, The Netherlands), ScienceON, and KoreaMed. The search spanned all available studies up to May 2025. All retrieved records were imported into Microsoft Excel, and duplicates were removed. Titles and abstracts were screened for eligibility, followed by full-text assessment of potentially relevant studies. Detailed search strategies and outcomes for each database are provided in Tables S1–S5. 

### 2.3. Study Selection

#### 2.3.1. Study Design

Only randomized controlled trials (RCTs) with a crossover design evaluating the effects of time-restricted eating (TRE) on glycemic control were included. Non-randomized trials and non-crossover designs were excluded to minimize bias. No restrictions were placed on the language of publication.

#### 2.3.2. Participants

Eligible studies included patients with type 2 diabetes or impaired fasting glucose (IFG). Studies were excluded if TRE was not clearly defined or if TRE was combined with other interventions. There were no restrictions regarding sex, ethnicity, or nationality.

#### 2.3.3. Interventions and Comparators

Included studies implemented TRE as the primary intervention for improving glycemic outcomes. Studies with non-restricted or conventional dietary patterns were accepted as control groups. Trials without a TRE-specific intervention were excluded.

#### 2.3.4. Outcome Measures

Primary outcomes included glycemic parameters such as HbA1c, fasting plasma glucose, and glucose area under the curve (AUC). Secondary outcomes included measures of glycemic variability or excursion.

### 2.4. Data Extraction

Data from included studies were extracted using a standardized Excel form, which had been pilot tested for consistency. Extracted variables included author name, country, publication year, sample size, participant demographics, details of the intervention and control, outcome measures, statistical methods, and results. Two reviewers independently performed the selection and extraction process. In cases of missing or unclear data, study authors were contacted for clarification where possible.

### 2.5. Risk of Bias Assessment

Two reviewers independently assessed the risk of bias (ROB) for each included study using the Cochrane Collaboration’s ROB tool covering six domains: (1) random sequence generation, (2) allocation concealment, (3) blinding of participants, (4) blinding of outcome assessors, (5) incomplete outcome data, and (6) selective reporting. Each domain was rated as low (L), high (H), or unclear (U) risk of bias.

### 2.6. Data Synthesis and Analysis

Statistical analyses were performed using RevMan 5.4 (The Nordic Cochrane Centre, Copenhagen, Denmark). For continuous outcomes, results were reported as mean differences with 95% confidence intervals (CIs). Sensitivity analyses were conducted to evaluate the impact of study quality, focusing on trials with a low risk of bias. Where heterogeneity precluded meta-analysis, a narrative synthesis of the findings was provided.

## 3. Results

### 3.1. Study Selection and Description

**Figure 1:**
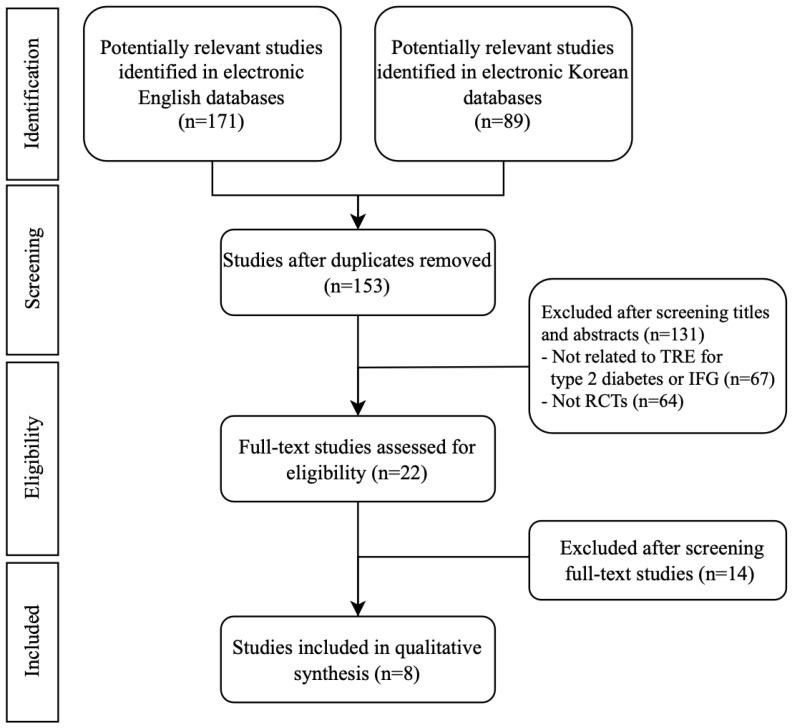
Flowchart of the RCT selection process. RCTs: randomized controlled trials.

A total of 154 studies were identified across five databases (three in English and two in Korean). After going through the identification, screening, and selection process, eight studies were ultimately included. Of these, three were conducted in Australia; two in the United States; and one each in Denmark, Thailand, and the Netherlands. Table 1 provides a summary of the key characteristics of the included studies. Table 2 outlines the specific TRE (time-restricted eating) and control groups’ protocols used in each study. Figure 1 illustrates the study selection process in a flow diagram following the PRISMA (Preferred Reporting Items for Systematic Reviews and Meta-Analyses) guidelines. A meta-analysis was conducted on six randomized controlled trials (RCTs), focusing on studies that implemented the same intervention protocol.

### 3.2. Study Population

A total of 312 participants were included. All participants were individuals diagnosed with type 2 diabetes or IFG, and all studies recruited participants regardless of sex.

### 3.3. Outcomes

The included studies reported a variety of outcome measures. Primary outcomes included blood glucose AUC, average glucose, fasting glucose, HbA1c, and postprandial 2 h blood glucose. The secondary outcome was the time in range (TIR) of blood glucose levels.

#### 3.3.1. Glucose AUC

Findings related to AUC were reported in studies by Evelyn B. Parr (2023, 2024) [19,25] and Jonas Salling Quist (2024) [23].

In Parr’s 2023 study, the postprandial 1 h and 2 h glucose AUCs were significantly reduced in the TRE group compared to the control group, suggesting that TRE effectively attenuates both the magnitude and rate of glucose elevation. The 2024 follow-up study reported total AUC values at 1, 2, 4, and 6 months. Significant reductions were observed at 1 and 6 months in the TRE group, while no notable differences were found at 2 and 4 months. Quist’s 2024 study also demonstrated a reduction in glucose AUC at 3 months in the TRE group, though the results were not statistically significant.

#### 3.3.2. Fasting Glucose

Fasting glucose was evaluated in studies by Evelyn B. Parr (2023) [19], Jonas Salling Quist (2024) [23], Charlotte Andriessen (2022) [21], Hegedus E (2024) [22], and Suthutvoravut U (2023) [20].

Parr’s study showed significantly lower fasting glucose levels in the TRE group compared to the control. Quist’s research reported significant reductions in fasting glucose at both 3 and 6 months in the TRE group. Andriessen (2022) [21] assessed fasting glucose after 11 h of fasting on day 21 and found a significant decrease in the TRE group, indicating that improvements in fasting glucose can occur even with short-term intervention. In contrast, Hegedus E’s (2024) [22] study observed a decrease in fasting glucose after TRE, but the change was not statistically significant. In the study by Suthutvoravut U (2023) [20], fasting plasma glucose levels decreased in the group that underwent 12 weeks of TRE.

#### 3.3.3. HbA1c

The effect of TRE on HbA1c was examined in studies by Jonas Salling Quist (2024) [23], Evelyn B. Parr (2024) [25], Hegedus E (2024) [22], Pavlou V (2023) [24], and Suthutvoravut U (2023) [20].

Quist reported a trend toward reduced HbA1c at 3 and 6 months in the TRE group, with only the 6-month result reaching statistical significance. Parr’s 2024 study analyzed HbA1c in both mmol/mol and percentage units, and found significant reductions in both metrics in the TRE group, highlighting the potential for TRE to improve glycemic control within a relatively short timeframe. Hegedus E’s study showed a slight reduction in HbA1c in the control group rather than in the TRE group, but this was not statistically significant. Pavlou V (2023) [24] demonstrated a significant reduction in HbA1c in the TRE group compared to the control, providing additional evidence for TRE’s long-term glycemic benefits. In the study by Suthutvoravut U (2023) [20], HbA1c levels also decreased in the group that underwent 12 weeks of TRE.

#### 3.3.4. Time in Range (TIR)

TIR within normoglycemic range was assessed in studies by Evelyn B. Parr (2023, 2024) [19,25], Hegedus E, and Pavlou V (2023) [22,24].

In Parr’s 2023 study, TIR significantly increased following TRE intervention, with participants spending approximately 2.5 more hours per day within the target glucose range. The 2024 follow-up study also reported improvements in TIR, suggesting that TRE may enhance glycemic stability. Pavlou V evaluated the percentage of time spent in the euglycemic range, which significantly increased in the TRE group, further supporting the role of TRE in improving the quality of glycemic control. Although TIR increased in Hegedus E’s study post-TRE, the changes were not statistically significant.

#### 3.3.5. Average (Mean) Glucose

Average glucose levels were reported in studies by Evelyn B. Parr (2024) [25], Hegedus E, and Pavlou V (2023) [22,24]. Parr’s study indicated a trend toward lower mean glucose levels in the TRE group, though statistical significance could not be determined due to reporting limitations. Nonetheless, a favorable trend was observed. In Hegedus E’s study, changes in mean glucose were noted, but they did not reach statistical significance. In contrast, Pavlou V’s study showed a significant reduction in average glucose in the TRE group compared to the control, suggesting that TRE contributes to improved glucose regulation.

### 3.4. Assessment for Risk of Bias

**Figure 2:**
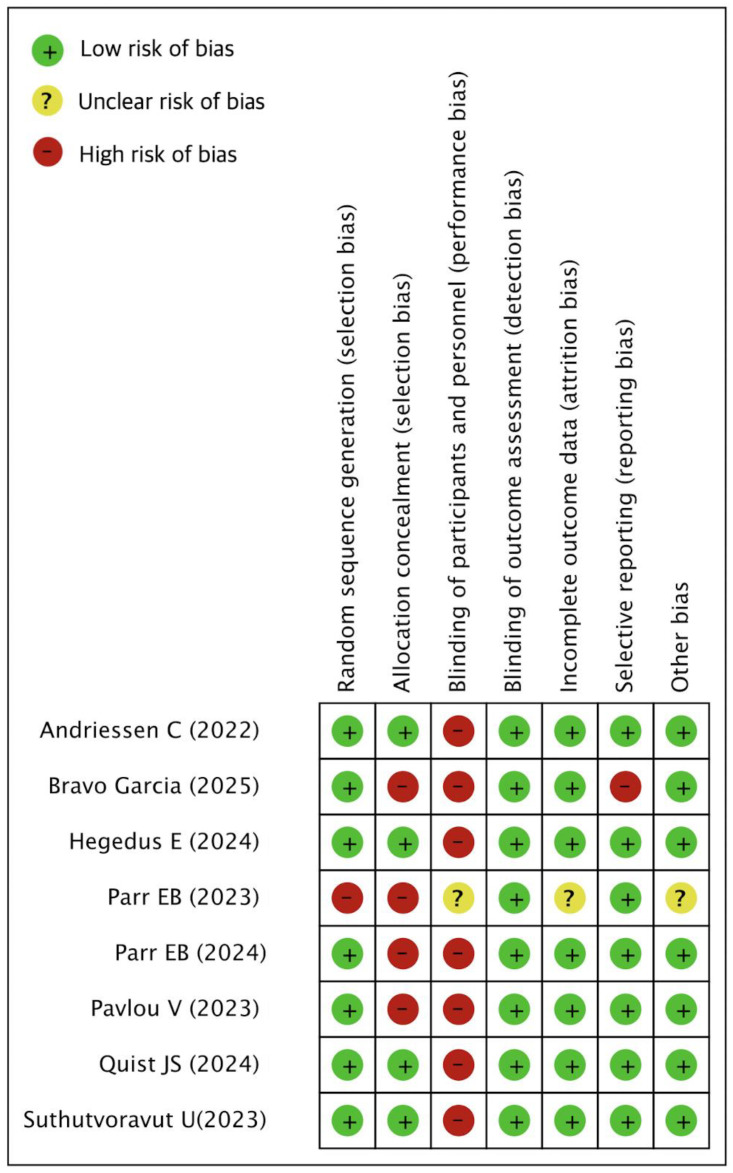
Risk of bias [16,19,20,21,22,23,24,25].

Figure 2 presents the risk of bias assessment for the included randomized controlled trials (RCTs), conducted using the Cochrane Risk of Bias tool. Performance bias across all studies was rated as low due to the inherent nature of the time-restricted eating (TRE) intervention. Because participants are typically aware of their eating schedules, blinding is impractical; thus, this domain was consistently judged to carry minimal influence on study validity. In the work by Bravo Garcia (2025) [16], the risk of selective reporting bias was rated as high. Although total energy intake was not prespecified in the original protocol, it was later incorporated into the analysis plan and trial registry. Given the broad scope of secondary outcomes, the potential for selective reporting of favorable results cannot be excluded, even though multiple comparisons were adjusted using the false discovery rate (FDR) method. For Parr EB (2023) [19], both random sequence generation and allocation concealment were rated as high risk due to the structural characteristics of the study. Specifically, the study was designed as a non-randomized pre-post intervention without a comparison group, rendering these domains inapplicable by design. As such, no allocation occurred, and concealment was not possible. In the same study, attrition bias was considered unclear. Although the number of participants completing the study was reported, details regarding the number and reasons for dropouts during the intervention phase were insufficient. Moreover, while missing continuous glucose monitoring (CGM) data were imputed, it was unclear whether analyses were conducted on an intention-to-treat (ITT) or per-protocol basis. Regarding other sources of bias, Parr EB (2023) [19] was judged to have unclear risk. The non-randomized design raises the possibility of structural confounding, such as self-reported dietary adherence, unmeasured lifestyle factors, and unknown intervention fidelity. Although the analyses included limited covariate adjustments (e.g., for HbA1c and sex), these were likely insufficient to account for inherent biases. Overall, aside from these noted exceptions, the remaining studies demonstrated low risk in domains related to randomization, outcome assessment, incomplete data, selective reporting, and other biases.

### 3.5. Meta-Analysis or Quantitative Analysis of the Include Articles

A meta-analysis was performed using data extracted from six studies that met the predefined inclusion criteria. The pooled outcomes focused on glycemic indices, including fasting glucose (mmol/mol), ΔHbA1c (%), and time in range (TIR) (%). Although five glycemic parameters were initially considered for analysis, mean glucose and glucose AUC were excluded from the meta-analysis due to insufficient data availability and inconsistent reporting formats across studies. The mean difference (MD) for each included outcome was calculated using a fixed-effects model, and heterogeneity across studies was assessed using the I^2^ statistic.

To ensure consistency across studies, all outcome variables were converted to absolute post-intervention values whenever original data were reported as change from baseline (Δ values). This standardization was essential for valid pooling in the meta-analyses, as several studies provided only Δ values without reporting post-intervention levels directly. The absolute mean values were calculated by adding the reported baseline mean and the mean change from baseline.

In cases where outcome variables were reported as negative changes (e.g., Δ values with negative signs), the values were converted to absolute post-intervention values to facilitate consistent interpretation across studies. This approach ensured that all pooled values reflected actual levels rather than directionality of change. Baseline values used for this transformation were as follows: Hegedus E (2024) [22]: Fasting glucose: 6.8 ± 1.0 (TRE), 6.4 ± 0.9 (control); Pavlou V (2023) [24]: HbA1c: 8.3 (TRE), 7.9 (control); TIR: 30 (TRE), 32 (control); mean glucose level: 180 (TRE), 177 (control). These baseline values were added to the reported changes to derive absolute post-intervention values, which were then used in the final meta-analytic models.

Standard deviations for the absolute values were calculated by combining the baseline standard deviation and the standard deviation of the change scores, assuming independence between the two. Specifically, the square root of the sum of their squared values was used.

When only the 95% confidence intervals (CIs) for Δ values were available, standard deviations were estimated using the following approximation: (upper CI–lower CI) divided by 3.92.

These conversions enabled harmonized input data for each meta-analysis and enhanced the interpretability and robustness of the pooled results. The converted values used for the meta-analyses are detailed in Supplementary File S2 (‘Delta Conversion’).

#### 3.5.1. Fasting Glucose

**Figure 3:**
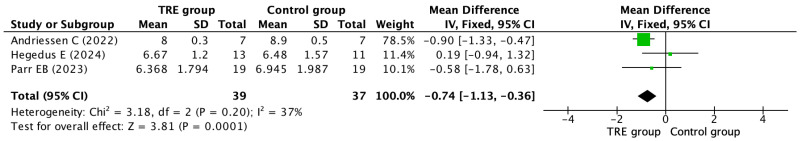
Meta-analysis of the effect of time-restricted eating (TRE) versus control on fasting glucose [19,21,22].

Three randomized controlled trials with a total of 76 participants (TRE: *n* = 39; control: *n* = 37) were included in this meta-analysis. Time-restricted eating (TRE) significantly improved the outcome compared to the control group (MD: −0.74; 95% CI: −1.13 to −0.36; *p* = 0.0001). Heterogeneity was low (I^2^ = 37%), indicating acceptable consistency among the studies (Figure 3).

However, a sensitivity analysis excluding the study by Hegedus E (2024) [22], which reported a non-significant result with a wide confidence interval (MD: 0.19 [−0.94, 1.32]), reduced heterogeneity to 0%. This suggests that the study by Hegedus may have introduced slight inconsistency due to its small sample size, imprecise estimate, and minimal effect size. In contrast, the remaining two studies consistently favored TRE, reinforcing its potential benefit in improving fasting glucose levels.

#### 3.5.2. ΔHbA1c

**Figure 4:**
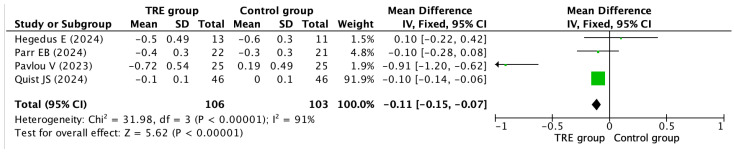
Meta-analysis of the effect of time-restricted eating (TRE) versus control on ΔHbA1c [22,23,24,25].

Four randomized controlled trials involving 209 participants (TRE: *n* = 106; control: *n* = 103) were included in this meta-analysis. Time-restricted eating (TRE) significantly improved the outcome compared to the control group (MD: −0.11; 95% CI: −0.15 to −0.07; *p* < 0.00001) (Figure 4). However, substantial heterogeneity was observed (I^2^ = 91%), indicating considerable variability among the included studies. This heterogeneity may be attributed to differences in study design, population characteristics, intervention duration, adherence levels, or measurement methods. Notably, while three studies reported a comparable effect size favoring time-restricted eating (TRE), the study by Pavlou V. (2023) [24] demonstrated a markedly larger effect (MD: −0.91), which disproportionately contributed to the observed heterogeneity (I^2^ = 91%). This divergence appears to stem from two key factors: first, the TRE group in Pavlou’s study had a substantially higher baseline HbA1c compared to the control group (8.3% vs. 7.9%), increasing the potential for larger absolute reductions; second, it was the only study in which HbA1c levels increased in the control group—a pattern not observed in the other trials, where control groups either maintained or slightly improved glycemic markers. This upward drift in the control arm may have exaggerated the apparent treatment effect by inflating the between-group difference in HbA1c change. The increase could plausibly be attributed to the absence of active dietary counseling or behavioral monitoring in the control group, which received no structured intervention beyond routine follow-up.

To assess the robustness of the pooled estimate, a sensitivity analysis was conducted excluding the Pavlou V. study. This analysis yielded a consistent pooled effect (MD: −0.10; 95% CI: −0.14 to −0.06) and eliminated heterogeneity entirely (I^2^ = 0%), indicating that the overall conclusion remains unchanged despite the initial variability. The corresponding forest plot is presented in Supplementary File S3 (Figure S1, ‘forest plot excluding the high-effect study’).

#### 3.5.3. Time in Range (TIR)

**Figure 5:**
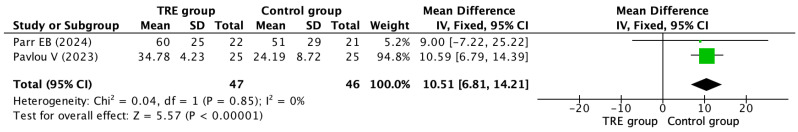
Meta-analysis of the effect of time-restricted eating (TRE) versus control on time in normoglycemic range [24,25].

Two randomized controlled trials including 93 participants (TRE: *n* = 47; control: *n* = 46) were included in this meta-analysis. Time-restricted eating (TRE) significantly improved the outcome compared to the control group (MD: 10.51; 95% CI: 6.81 to 14.21; *p* < 0.00001) (Figure 5). Heterogeneity was negligible (I^2^ = 0%), indicating strong consistency between the two studies. Although the study by Parr EB (2024) [25] showed a wide confidence interval with lower precision due to the small sample size and variability, the effect direction was consistent. Both studies favored the TRE group, supporting the effectiveness of TRE in enhancing the measured outcome.

## 4. Discussion

This study is significant in that it is the first to statistically confirm the effects of time-restricted eating (TRE) on glycemic control specifically in patients with diabetes. Previous research on TRE primarily focused on individuals with obesity or the general population, and while some randomized controlled trials (RCTs) have been conducted in diabetic patients, their findings have been inconsistent. This meta-analysis thus holds added value by consolidating and clarifying the glycemic effects of TRE in a diabetic cohort.

The analysis included eight studies from various countries, including Australia, the United States, Denmark, the Netherlands, and Thailand, encompassing a total of 312 participants—all of whom were diagnosed with diabetes or IFG [16,19,20,21,22,23,24,25]. Of these, six studies provided sufficient data for quantitative synthesis and were included in the meta-analysis. While five glycemic outcomes were initially considered, only three—fasting glucose, HbA1c, and time in range (TIR)—were analyzed quantitatively due to inconsistent reporting formats and missing post-intervention data for mean glucose and glucose AUC. No stratification by age was performed, and both CGM (continuous glucose monitoring) and non-CGM methods were employed across studies. The intervention consistently compared TRE with either standard diets or no dietary intervention.

The meta-analysis showed that TRE significantly reduced fasting glucose by −13.8 mg/dL (equivalent to −0.74 mmol/L; 95% CI: −21.2 to −6.4, *p* = 0.001), HbA1c by −0.11% (95% CI: −0.15 to −0.07, *p* < 0.001), and increased TIR by +10.5% (95% CI: 6.8 to 14.2, *p* < 0.001). Although the reduction in HbA1c was modest, it is considered clinically meaningful in the context of short-term behavioral interventions. Consistent improvements in both fasting glucose and HbA1c suggest that TRE may serve as a potential dietary strategy for enhancing glycemic control over both short- and long-term periods [16,19,20,21,23,24,25]. Notably, the improvement in TIR, with minimal heterogeneity across studies, underscores the robustness of TRE’s effect on glycemic stability. Furthermore, several studies reported that participants found TRE more sustainable and easier to adhere to than conventional caloric restriction or structured diabetic meal planning. This suggests that, beyond its metabolic benefits, TRE may offer a feasible and acceptable long-term dietary approach for individuals with diabetes [19,24,25].

Fasting glucose levels were significantly reduced by TRE, likely due to enhanced insulin sensitivity and reduced hepatic glucose production [19,21,22]. Positive effects were also observed in HbA1c and ΔHbA1c [19,23,24]. Most included studies consistently demonstrated a trend toward reduced glycemic exposure, suggesting that TRE may help attenuate postprandial glucose excursions.

TRE’s impact on glycemic indices may be partly mediated through circadian rhythm alignment [26]. While the central circadian pacemaker resides in the suprachiasmatic nucleus (SCN) of the brain, peripheral clocks exist in organs such as the liver, pancreas, and adipose tissue [26]. TRE can resynchronize these peripheral clocks to feeding–fasting rhythms, thereby promoting metabolic homeostasis [26].

During fasting, increased AMP/ATP ratio activates AMPK, which promotes degradation of the clock protein Cry1 and activates SIRT1, a NAD^+^-dependent histone deacetylase. SIRT1 binds to the Clock:Bmal1 complex and suppresses Per2 expression, thereby strengthening circadian gene rhythms [21,27,28]. These processes downregulate hepatic gluconeogenesis genes (e.g., pyruvate carboxylase, glucose-6-phosphatase) and upregulate glucokinase, collectively contributing to improved glycemic control [21,27,28].

Importantly, these metabolic improvements were found to occur independently of weight loss and were shown to enhance β-cell function regardless of changes in body weight. Additionally, the observed benefits were not associated with changes in lipid content, insulin sensitivity, mitochondrial function, or overall energy metabolism, suggesting that TRE alone can meaningfully improve glycemic indices.

TIR within the normoglycemic range was significantly higher in the TRE groups compared to controls [19,22,24,25]. This suggests that TRE not only reduces blood glucose but also promotes stability within the desired glycemic range, which may reduce glucose variability and mitigate the risk of hyper- and hypoglycemic episodes. These findings highlight the potential of TRE in enhancing condition management and quality of life for diabetic patients. Notably, this was the only glycemic index with no between-study heterogeneity, implying that TRE’s stabilizing effect on glycemia is robust and reproducible across diverse populations and settings.

TIR has been recognized by the American Diabetes Association as a key metric for diabetes management in patients using CGM and is known to correlate strongly with HbA1c [29]. Studies have shown that every 10% reduction in TIR is associated with a 64% increased risk of retinopathy progression and a 40% increased risk of microalbuminuria [30]. These findings underscore the clinical relevance of TIR not only in glycemic control but also in the prevention of diabetes-related complications. Thus, TRE may offer a lifestyle intervention that contributes to both glycemic stability and long-term outcomes in patients with diabetes.

Nevertheless, several limitations should be noted. First, due to the nature of dietary interventions, double-blinding was not feasible, and placebo effects could not be entirely excluded. Second, substantial heterogeneity was observed in the meta-analysis of ΔHbA1c (I^2^ = 91%), largely driven by a single study with imbalanced baseline characteristics and control group deterioration. Sensitivity analysis excluding this study eliminated heterogeneity (I^2^ = 0%), supporting the robustness of the pooled estimate. Third, glucose monitoring methods varied, with some studies using CGM and others relying on single-point plasma glucose measurements, potentially limiting the comparability of outcomes. Moreover, because we separated change from baseline values from absolute values and only included studies that met strict inclusion criteria, meta-analyses could be conducted for fasting glucose, ΔHbA1c, and TIR only. As a result, a comprehensive meta-analysis across all reported outcomes was not feasible.

Despite these limitations, this study provides systematic evidence that TRE may significantly improve glycemic control in individuals with diabetes. Future high-quality RCTs with standardized TRE protocols and long-term follow-up are warranted to validate and extend these findings.

## 5. Conclusions

This meta-analysis provides the first comprehensive statistical synthesis of time-restricted eating (TRE) effects on glycemic control specifically in patients with diabetes.

The findings demonstrate that TRE significantly improves fasting glucose, HbA1c, and time in range (TIR), as confirmed by meta-analysis. Additional glycemic indices such as mean glucose and glucose AUC showed favorable trends in individual studies but were not included in the quantitative synthesis due to heterogeneity in reporting. Notably, TRE was associated with consistent reductions in fasting glucose and HbA1c and a robust increase in TIR with no observed heterogeneity, suggesting high replicability and potential for clinical utility.

Mechanistically, these benefits may be attributed to enhanced insulin sensitivity and synchronization of peripheral circadian clocks through feeding–fasting cycles rather than changes in body weight or energy metabolism alone. The favorable adherence profile reported in several studies also highlights TRE’s promise as a sustainable dietary strategy for diabetes management.

Despite limitations such as methodological heterogeneity and the lack of double-blinding, the collective evidence supports TRE as an effective, non-pharmacological intervention for improving both short- and long-term glycemic regulation. Further high-quality randomized controlled trials with standardized intervention protocols and extended follow-up are warranted to establish TRE as a guideline-recommended therapeutic option in diabetes care.

## Figures and Tables

**Table 1:** TRE and control groups’ intervention protocols.

Study (Author, Year)	TRE Group	Control Group	Duration
**Evelyn B. Parr** ** (2023)** [19]	9 h eating window (10:00–19:00), 4 weeks, after 2-week habitual monitoring	Normal meal	4 weeks
**Bravo-Garcia AP** ** (2025)** [16]	Eating window: 10:00–18:00 + exercise (13 sessions)	Eating window: 08:00–20:00 + exercise (11 sessions)	14 h
**Jonas Salling Quist** ** (2024)** [23]	Self-selected 10 h eating window between 06:00–20:00	Habitual living for 3 months	3 months
**Charlotte Andriessen** ** (2022)** [21]	Standardized meal at 16:40; fasting from 17:00	Standardized meal at 20:40; fasting from 21:00	3 months
**Evelyn B. Parr** ** (2024)** [25]	Eating window: 10:00–19:00 as often as possible during intervention	Publicly available nutrition guidance + dietary discussion	6 months
**Hegedus E** ** (2024)** [22]	Eating window: 12:00–20:00 (±1 h), 7 days/week	Eating window ≥ 12 h/day, 7 days/week	12 weeks
**Pavlou V (2023)** [24]	Eating window: 12:00–20:00 without calorie counting	(1) Caloric restriction (−25% baseline intake); (2) usual habits (weight maintenance, usual eating/exercise)	6 months
**Suthutvoravut U (2023)** [20]	Eating window: 08:00–17:00, 15 h fasting; with/without behavioral economics (BE)	Usual care (no TRE), no behavioral incentive or reminders	12 weeks

**Table 2:** Details of included studies.

Author (Year)	Nation	Participants	Sample Size	Duration	Experimental Group (No. of Participants Analyzed)	Control Group (No. of Participants Analyzed)	Outcome Measures	Result of Experimental Group	Result of Control Group	Assessment
Evelyn B. Parr (2023) [19]	Australia	T2DM	19	6 weeks	TRE (19)	Control (19)	(1) TIR (%) (2) TIR (hours) (3) blood glucose AUC (mmol/Lxh) (4) Fasting glucose(mmol/mol)	10 ± 18% 2.5 ± 4.2h −0.7 ± 0.4 6.368 ± 1.794	- - - 6.945 ± 1.987	Positive
Bravo-Garcia AP (2025) [16]	Australia	T2DM	24	4 conditions × 2-day trials with 3- to 7-day washouts in between	TRE + exercise (13)	Control + exercise (11)	(1) Postprandial 2 h plasma glucose after lunch (mmol/Lx2h) (2) Postprandial 2 h plasma glucose after dinner (mmol/Lx2h)	−131 ± 129 −208 ± 124	- -	Positive
Jonas Salling Quist (2024) [23]	Denmark	T2DM	92	3 months	TRE (46)	Control (46)	(1) Δ 3 months HbA1c (mmol/mol) (2) Δ 6 months HbA1c (mmol/mol) (3) Δ 3 months fasting glucose (mmol/L) (4) Δ 6 months fasting glucose (mmol/L) (5) Δ 3 months glucose AUC (mmol/min)	−1 (−1 to −0) 0 (−1 to 0) −0.1 (−0.2 to 0.0) 0.0 (−0.1 to 0.2) −66.7 (−101.2 to −321)	0 (−0 to 1) 0 (−1 to 1) 0 (−0.1 to 0.1) 0.1 (−0.1 to 0.3) 2.1 (−31.1 to 35.2)	Positive
Charlotte Andriessen (2022) [21]	The Netherlands	T2DM	14	3 weeks	TRE (7)	Control (7)	Fasting glucose (mmol/mol)	8.0 ± 0.3	8.9 ± 0.5	Positive
Evelyn B. Parr (2024) [25]	Australia	T2DM	43	6 months	TRE (22)	Diet (21)	(1) ΔHbA1c (mmol/mol) (2) ΔHbA1c (%) (3) 1 month total AUC (4) 2 months total AUC (5) 4 months total AUC (6) 6 months total AUC (7) TIR (%) (8) Mean glucose level (mmol/L)	−5 (−8 to 0) −0.4 (−0.7 to 0.0) 7.8 ± 1.4 8.4 ± 1.8 8.2 ± 1.5 7.6 ± 1.4 60 ± 25 Δ −0.8	−4 (−7 to 1) −0.3 (−0.6 to 0.1) 8.0 ± 1.7 7.7 ± 1.4 8.2 ± 1.6 8.3 ± 2.0 51 ± 29	Positive
Hegedus E (2024) [22]	USA	T2DM	17	3 months	TRE (8)	Control (9)	(1) Δ Average glucose (mmol/L) (2) Δ TIR (%) (3) Fasting glucose (mmol/mol)	0.067 (−1.167, 1.278) 3.6(−8, 15) 6.67 ± 1.2	0.056 (−0.833, 0.889) −1.7 (−0.2, 9) 6.48 ± 1.57	NSD
		T2DM	24	3 months	TRE (13)	Control (11)	(4) ΔHbA1c (%)	−0.5 (−0.9, −0.01)	−0.6 (−0.9, −0.2)	
Pavlou V (2023) [24]	USA	T2DM	50	6 months	TRE (25)	Control (25)	(1) ΔHbA1C (%) (2) ΔTIR (%) (3) ΔMean glucose level (mmol/mol)	−0.72 (−1.25, −0.18) 4.78 (−3.52, 13.08) −0.612 (−1.381, 0.157)	0.19 (−0.30, 0.68) −7.81 (−24.90, 9.27) 1.751 ( −130.252, 3.753)	Positive
Suthutvoravut U (2023) [20]	Thailand	IFG	46	3 months	TRE (24)	Control (22)	(1) Fasting plasma glucose (mmol/mol) (2) HbA1c (%)	−0.263 (−0.477, −0.050) ^†^ −0.24 (−0.457, −0.03) ^†^	- -	Positive

AUC: Area under the curve; NSS: Not statistically significant; NSD: Not significantly different; IFG: Impaired fasting glucose; T2DM: Type 2 diabetes mellitus; TIR: Time in range; Δ indicates a value representing change from baseline; † indicates a value compared to the control group.

## Data Availability

Data supporting the results of this study are available from the original published articles included in the meta-analysis.

## Supplementary Materials

The following supporting information can be downloaded at: https://www.mdpi.com/article/10.3390/ijms26157310/s1.

## Abbreviations

The following abbreviations are used in this manuscript:

AUC	Area under the curve
NSS	Not statistically significant
IFG	Impaired fasting glucose
T2DM	Type 2 diabetes mellitus
TIR	Time in range

## References

[1] Zheng Y., Ley S.H., Hu F.B. (2018). Global Aetiology and Epidemiology of Type 2 Diabetes Mellitus and Its Complications. Nat. Rev. Endocrinol..

[2] Banerjee M., Khursheed R., Yadav A.K., Singh S.K., Gulati M., Pandey D.K., Prabhakar P.K., Kumar R., Porwal O., Awasthi A. (2020). A Systematic Review on Synthetic Drugs and Phytopharmaceuticals Used to Manage Diabetes. Curr. Diabetes Rev..

[3] Kolb H., Martin S. (2017). Environmental/Lifestyle Factors in the Pathogenesis and Prevention of Type 2 Diabetes. BMC Med..

[4] Eckel R.H., Bornfeldt K.E., Goldberg I.J. (2021). Cardiovascular Disease in Diabetes, beyond Glucose. Cell Metab..

[5] Cummings J., Ortiz A., Castellino J., Kinney J. (2022). Diabetes: Risk Factor and Translational Therapeutic Implications for Alzheimer’s Disease. Eur. J. Neurosci..

[6] Lei C., Duan J., Ge G., Zhang M. (2021). Association between Neonatal Hyperglycemia and Retinopathy of Prematurity: A Meta-Analysis. Eur. J. Pediatr..

[7] Kozakova M., Palombo C. (2016). Diabetes Mellitus, ArterialWall, and Cardiovascular Risk Assessment. Int. J. Env. Res. Public Health.

[8] Shikata K., Ninomiya T., Kiyohara Y. (2013). Diabetes Mellitus and Cancer Risk: Review of the Epidemiological Evidence. Cancer Sci..

[9] ElSayed N.A., Aleppo G., Aroda V.R., Bannuru R.R., Brown F.M., Bruemmer D., Collins B.S., Cusi K., Das S.R., Gibbons C.H. (2023). Summary of Revisions: Stand. Care Diabetes—2023. Diabetes Care.

[10] (2024). Trends in the prevalence of diabetes, 2013–2022. Public Health Weekly Rep..

[11] Termannsen A.-D., Varming A., Hansen G.S., Bjerre N., Persson F., Bagger J.I., Hansen D.L., Ewers B., Jørgensen N.B., Blond M.B. (2025). Time-Restricted Eating Is a Feasible Dietary Strategy in the Treatment of Complicated Type 2 Diabetes: The RESET2 Pilot Study. J. Nutr. Educ. Behav..

[12] Chang Y.J., Turner L., Teong X.T., Zhao L., Variji A., Wittert G.A., Thompkins S., Vincent A.D., Grosser L., Young M.J. (2025). Comparing the Effectiveness of Calorie Restriction with and without Time-Restricted Eating on the Circadian Regulation of Metabolism: Rationale and Protocol of a Three-Arm Randomised Controlled Trial in Adults at Risk of Type 2 Diabetes. Nutr. Res..

[13] Dehghani S., Karimi P., Tarei N.N., Masoumvand M., Manesh M.A.N., Ramezani E., Askari V.R. (2025). Comparison of the Effect of Intermittent Fasting with Mediterranean Diet on Glycemic, Lipid, and Anthropometric Indices in Type 2 Diabetes: A Review of Randomized Controlled Trials. Curr. Hypertens. Rev..

[14] Lin S., Cienfuegos S., Ezpeleta M., Pavlou V., Corapi S., Runchey M.-C., Alexandria S.J., Tussing-Humphreys L., Varady K.A. (2025). Time-Restricted Eating Versus Daily Calorie Restriction: Effects on Inflammatory Markers over 12 Months in Adults with Obesity. Nutrients.

[15] Catenacci V.A., Ostendorf D.M., Pan Z., Kaizer L.K., Creasy S.A., Zaman A., Caldwell A.E., Dahle J., Swanson B., Breit M.J. (2025). The Effect of 4:3 Intermittent Fasting on Weight Loss at 12 Months. Ann. Intern. Med..

[16] Bravo-Garcia A.P., Radford B.E., Hall R.C., Broome S.C., Tee N., Arthur B., Janssens K., Johnston R.D., Halson S.L., Devlin B.L. (2025). Combined Effects of Time-Restricted Eating and Exercise on Short-Term Blood Glucose Management in Individuals with Type 2 Diabetes Mellitus: The TREx Study, a Randomised Controlled Trial. Diabetes Res. Clin. Pract..

[17] Braun L., Haumann H., Polanc A., Koch R., Feil E., Klein A., Salm C., Peters-Klimm F., Hübner G., Thies C. (2025). Time-Restricted Eating (TRE) for Obesity in General Practice: Study Protocol of a Controlled, Randomized Implementation Study (INDUCT) within the Research Practice Network Baden-Wuerttemberg (FoPraNet-BW). Nutr. J..

[18] Huang X., Huang G., Wei G. (2025). Intermittent Fasting for Glycemic Control in Patients with Type 2 Diabetes: A Meta-Analysis of Randomized Controlled Trials. Nutr. Hosp..

[19] Parr E.B., Steventon-Lorenzen N., Johnston R., Maniar N., Devlin B.L., Lim K.H.C., Hawley J.A. (2023). Time-Restricted Eating Improves Measures of Daily Glycaemic Control in People with Type 2 Diabetes. Diabetes Res. Clin. Pract..

[20] Suthutvoravut U., Anothaisintawee T., Boonmanunt S., Pramyothin S., Siriyothin S., Attia J., McKay G.J., Reutrakul S., Thakkinstian A. (2023). Efficacy of Time-Restricted Eating and Behavioral Economic Intervention in Reducing Fasting Plasma Glucose, HbA1c, and Cardiometabolic Risk Factors in Patients with Impaired Fasting Glucose: A Randomized Controlled Trial. Nutrients.

[21] Andriessen C., Fealy C.E., Veelen A., van Beek S.M.M., Roumans K.H.M., Connell N.J., Mevenkamp J., Moonen-Kornips E., Havekes B., Schrauwen-Hinderling V.B. (2022). Three Weeks of Time-Restricted Eating Improves Glucose Homeostasis in Adults with Type 2 Diabetes but Does Not Improve Insulin Sensitivity: A Randomised Crossover Trial. Diabetologia.

[22] Hegedus E., Vu M.H., Salvy S.J., Bakhsh J., Goran M.I., Raymond J.K., Espinoza J.C., Vidmar A.P. (2024). Randomized Controlled Feasibility Trial of Late 8-Hour Time-Restricted Eating for Adolescents With Type 2 Diabetes. J. Acad. Nutr. Diet..

[23] Quist J.S., Pedersen H.E., Jensen M.M., Clemmensen K.K.B., Bjerre N., Ekblond T.S., Uldal S., Størling J., Wewer Albrechtsen N.J., Holst J.J. (2024). Effects of 3 Months of 10-h per-Day Time-Restricted Eating and 3 Months of Follow-up on Bodyweight and Cardiometabolic Health in Danish Individuals at High Risk of Type 2 Diabetes: The RESET Single-Centre, Parallel, Superiority, Open-Label, Randomised Controlled Trial. Lancet Healthy Longev..

[24] Pavlou V., Cienfuegos S., Lin S., Ezpeleta M., Ready K., Corapi S., Wu J., Lopez J., Gabel K., Tussing-Humphreys L. (2023). Effect of Time-Restricted Eating on Weight Loss in Adults With Type 2 Diabetes. JAMA Netw. Open.

[25] Parr E.B., Radford B.E., Hall R.C., Steventon-Lorenzen N., Flint S.A., Siviour Z., Plessas C., Halson S.L., Brennan L., Kouw I.W.K. (2024). Comparing the Effects of Time-Restricted Eating on Glycaemic Control in People with Type 2 Diabetes with Standard Dietetic Practice: A Randomised Controlled Trial. Diabetes Res. Clin. Pract..

[26] Regmi P., Heilbronn L.K. (2020). Time-Restricted Eating: Benefits, Mechanisms, and Challenges in Translation. iScience.

[27] Parr E.B., Devlin B.L., Hawley J.A. (2022). Perspective: Time-Restricted Eating—Integrating the What with the When. Adv. Nutr..

[28] Sutton E.F., Beyl R., Early K.S., Cefalu W.T., Ravussin E., Peterson C.M. (2018). Early Time-Restricted Feeding Improves Insulin Sensitivity, Blood Pressure, and Oxidative Stress Even without Weight Loss in Men with Prediabetes. Cell Metab..

[29] Battelino T., Danne T., Bergenstal R.M., Amiel S.A., Beck R., Biester T., Bosi E., Buckingham B.A., Cefalu W.T., Close K.L. (2019). Clinical Targets for Continuous Glucose Monitoring Data Interpretation: Recommendations From the International Consensus on Time in Range. Diabetes Care.

[30] Beck R.W., Bergenstal R.M., Riddlesworth T.D., Kollman C., Li Z., Brown A.S., Close K.L. (2019). Validation of Time in Range as an Outcome Measure for Diabetes Clinical Trials. Diabetes Care.

