# Supplementary material for: Time-Restricted Eating Improves Glycemic Control in Patients with Type 2 Diabetes: A Meta-Analysis and Systematic Review
Source: Int J Mol Sci. 2025 Jul 29;26(15):7310. doi: 10.3390/ijms26157310 (PMC12346854; doi:10.3390/ijms26157310)
Supplement: Supplementary file 1 [file ijms-26-07310-s001.zip › File S1_search strategy_meal window.pdf]

Table S1. Search strategy in Pubmed

NO.	Search strategy	Item
#1	diabetes mellitus OR type 2 diabetes OR type 1 diabetes	698.830
#2	eating window OR meal window	930
#3	glycemic control OR blood glucose OR glucose variability OR HbA1c	413,586
#4	Randomized Controlled Trial	866,997
#5	#1 AND #2 AND #3 AND #4	18

Table S2. Search strategy in Cochrane library

NO.	Search strategy	Item
#1	(diabetes mellitus OR type 2 diabetes OR type 1 diabetes)	114,595
#2	(eating window OR meal window)	408
#3	(glycemic control OR blood glucose OR glucose variability OR HbA1c)	86,568
#4	Randomized Controlled Trial	1,140,526
#5	#1 AND #2 AND #3 AND #4	64

Table S3. Search strategy in ScienceOn

NO.	Search strategy	Item
#1	전체=TRE|"Time restricted eating"|"시간 제한 식사"	214.302
#2	전체=diabetes|"glycemic control"|"blood sugar"|"blood glucose"	172,189
#3	전체=randomized controlled trial	418,521
#4	#1 AND #2 AND #3	40

전체: All; 시간 제한 식사: Time restricted eating.

Table S4. Search strategy in EMBASE

**NO.**	**Search strategy**	**Item**
#1	('diabetes mellitus'/exp OR 'type 2 diabetes mellitus'/exp OR 'type 1 diabetes mellitus'/exp OR 'diabetes mellitus':ti,ab OR 'type 2 diabetes':ti,ab OR 'type 1 diabetes':ti,ab OR diabetic*:ti,ab)	1,557,598
#2	('meal timing':ti,ab OR 'nutrient timing':ti,ab OR 'intake timing':ti,ab OR 'eating timing':ti,ab OR 'time of intake':ti,ab OR 'food timing':ti,ab OR 'temporal eating pattern':ti,ab)	1198
#3	('glycemic control':ti,ab OR 'glycaemic control':ti,ab OR 'blood glucose':ti,ab OR 'glucose variability':ti,ab OR 'hbA1c':ti,ab)	271,578
#4	#1 AND #2 AND #3	89

Table S5. Search strategy in Koreamed

**NO.**	**Search strategy**	**Item**
#1	TRE OR Time restricted eating OR eating window	12
#2	Diabetes OR glycemic control OR HbA1c OR blood glucose OR glucose	1984

NO.	Search strategy	Item
#3	#1 AND #2.	0
