# Supplementary material for: Time-Restricted Eating Improves Glycemic Control in Patients with Type 2 Diabetes: A Meta-Analysis and Systematic Review
Source: Int J Mol Sci. 2025 Jul 29;26(15):7310. doi: 10.3390/ijms26157310 (PMC12346854; doi:10.3390/ijms26157310)
Supplement: Supplementary file 1 [file ijms-26-07310-s001.zip › File S2_delta conversion.pdf]

Conversion of Δ Values to Absolute Values in Meta-Analysis

To ensure consistency across studies, all outcome variables were converted to absolute post-intervention values whenever original data were reported as change-from-baseline (Δ values). This standardization was essential for valid pooling in the meta-analyses, as several studies provided only Δ values without reporting post-intervention levels directly. The absolute mean values were calculated using the formula:

Mean_absolute = Mean_baseline + ΔMean

Standard deviations for the absolute values were computed by combining the reported baseline SD and the SD of the Δ values, assuming independence, using:

SD_absolute = sqrt((SD_baseline)^2 + (SD_Δ)^2)

When only the 95% confidence intervals (CIs) of Δ values were available, SDs were estimated as:

SD ≈ (Upper CI - Lower CI) / (2 × 1.96)

These conversions enabled harmonized input data for each meta-analysis and enhanced the interpretability and robustness of the pooled results. The converted values used for the meta-analyses are detailed in Supplementary Table X.
