# Supplementary figures and images for: Time-Restricted Eating Improves Glycemic Control in Patients with Type 2 Diabetes: A Meta-Analysis and Systematic Review
Source: Int J Mol Sci. 2025 Jul 29;26(15):7310. doi: 10.3390/ijms26157310 (PMC12346854; doi:10.3390/ijms26157310)

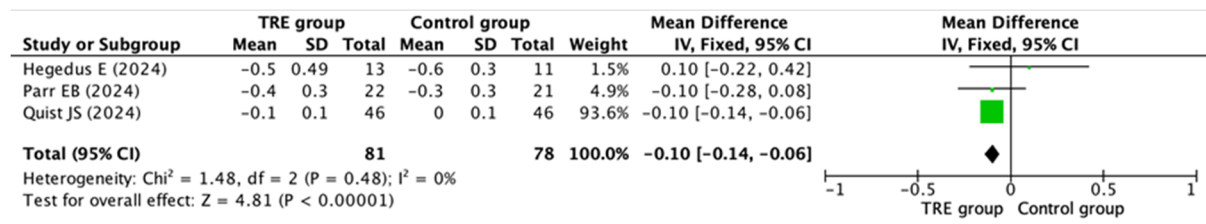

**Figure S1.** forest plot excluding the high-effect study [22,23,25].

Supplement: Supplementary file 1 [file ijms-26-07310-s001.zip › File S3_forest plot excluding the high-effect study.pdf]
